# Sexual Hypochondriasis

**Published:** 1879-10-20

**Authors:** F. J. Bumstead


### Southern Medical Record.

EDITORS:

T. S. POWELL, M.D. W. T. GOLDSMITH, M.D. R. C. WORD, M.D.

*R. C. WORD, M.D., Managing Editor.*

☞All Communications and Letters on Business connected with the RECORD must be addressed to the Managing Editor.

VOL. IX. ATLANTA, GA., OCTOBER 20, 1879. NO. 10.

### ORIGINAL AND SELECTED ARTICLES.

### *SEXUAL HYPOCHONDRIASIS.*

BY F. J. BUMSTEAD, M.D.

No small proportion of the patients who apply at the office of the venereal specialist, are afflicted only with hypochondriasis relating either to the appearance or the functions of their genital organs. These patients may be divided into two classes—first those who are ignorant of what the appearances of the genital organs normally are, or how far these appearances vary in sound persons, or who are ignorant of the influences which affect the functions of these organs in all men, even the most healthy. This class of patients, if blessed with common sense and confidence in their medical adviser, need only information to set them all right. But there is a second class of such patients, unfortunately the more numerous, whose minds are really unsound in reference to their sexual organs, who are unwilling to accept the statement of their physician that there is nothing the matter with them; who go on brooding over their imaginary trouble; who fall the ready victims of quacks; and who, after leading a miserable existence, a burden to themselves and their friends, sometimes become the inmates of a lunatic asylum or seek a suicide's death. If such patients cannot be made to listen to reason, and a manly spirit cannot be roused up in them, there is no hope for them, for neither medicine nor surgery can cure them.

I propose to mention some of the grounds of complaints with the subjects of sexual fear, or hypochondriasis, most commonly set forth to their physician.

With some the complaint is almost ludicrous, as for instance that one testicle hangs lower than the other—a condition which prevails with the great majority of men. Or the patient thinks that his penis or testicles are smaller than they ought to be, even when they are of very fair dimensions; or he complains of an itching or crawling sensation in the parts, which is not strange while his thoughts are constantly directed upon them. Again it is the cheesy excretion which forms in the furrow at the base of the glans; a few herpetic vesicles appearing from time to time, or a slight eczema of the penis, or the eczema marginatum which is so often developed in the inguinal fold, that makes him unhappy.

A prominent professional man applied to me a few years ago for a little follicular abscess on the sheath of the penis, which he kept open by constantly picking at it. His mind was perfectly clear on every other subject, but was insane on this. He imagined he had syphilis, and had communicated it to his wife and children. After a few months he committed suicide.

Again, enlargement of the scrotal veins, or varicocele, is a fruitful source of terror to many men. This, indeed, may exist to such an extent as to seriously incommode the patient, and to demand surgical interference; but, in a moderate degree, it is of trivial moment, and may be relieved by wearing a suspensory bandage.

But nocturnal emissions are the complaint of most of the subjects of sexual hypochondriasis, and this they will probably ascribe to masturbation, which they ignorantly practiced for a time in former years, until they had visited one of the vile "museums of anatomy," which infest our cities, and, either there or elsewhere, had read the terrible pictures of the dire effects of this habit, which quacks are wont to conjure up. Their emissions did not occur until after they had abandoned self-abuse, and hence, with illogical reasoning, they "must be due to that practice." Even men in adult life, who have been left widowers after years of unimpaired sexual power, will ascribe nocturnal emissions—due to their present continence—to early indiscretion.

The subjects of nocturnal emissions will tell you that, after each emission, they feel weak and exhausted; that they have pain in the back; and that they find their memory failing. They are apt to imagine also that the natural moisture of the urethra is semen; that the viscid fluid which oozes from the canal on sexual excitement is semen, and that they pass semen on straining at stool or in their urine—the latter being shown by some shreds which it contains when first passed, or by the sediment which is formed on standing.

Now, these men are to be told some plain truths. Nocturnal emissions occur independently of the practice of masturbation. Some of the most frequent cases I have ever seen have been in men who had never committed self-abuse. They are incident to early manhood, especially between the ages of fifteen and thirty, and less so as life advances. At this period the genital functions are most active; the secretion of the semen is constantly going on and must find vent somewhere, like a loaded rectum or a distended bladder. For a man in the prime of life and living continently, not to have an occasional nocturnal emission, is a rare exception. The frequency of these emissions will vary consistently with health, and will depend somewhat upon the purity of the thoughts of the individual, and upon whether the sexual desires have already been excited as by masturbation, illicit sexual intercourse, or the marriage state. Hence masturbators and widowers will be more exposed to them than those who have been continent from their youth up. With regard to their frequency, it may be said in general that once a month or once a fortnight is most common, but they may take place as often as two or three times a week without detriment to the health. They are very apt to occur in groups; and this is a point to be mentioned to patients, *i. e.*, he may be free from them for several weeks, and then will have two or three on successive nights or the same night.

In ninety-nine cases out of one hundred, these emissions require no medical or surgical treatment. The chief danger from them lies in the patient attaching undue importance to them, in dwelling upon them, and making himself miserable over them. If he can be induced to give his mind and body pure thoughts and healthy exercise, and to look upon their occurrence as a physical necessity, nature will take care of the rest.

It is now many years ago since a young man who, like most young men, had not been entirely free from self-abuse, picked up a pamphlet written by the late Dr. Bell, superintendent of the McLean Insane Asylum, in which the sin and degradation and the evils of masturbation were set forth. He at once abandoned the habit, but nocturnal emissions occurred and became a terror to him. He called upon Dr. Bell, and his first words, after announcing his mission, were to thank him for writing the pamphlet he had read. To his surprise, Dr. Bell replied that he was sorry he had ever written it; that the first edition was exhausted, and that he would never allow another to be published. "Why?" "Because I believe that what I said of the posslble evils of masturbation and nocturnal emissions was overdrawn and has done more harm by the fear it has excited in young men, than a continuance of the practice itself would have done." "Well! what medicine shall I take?" "No medicine whatever." (Second surprise of the young man.) "I shall only lay down some hygienic rules for you to follow. You must expect your nocturnal emissions to occur from time to time, but must not mind them. They will be less and less frequent as you grow older, and if you ever get married they will cease."

The young man had strength of mind enough to appreciate this advice and follow it. He found every word of the doctor's prognosis in his case to come true; and in hundreds of such cases which he afterwards met with in practice, when himself a physician, the same advice, when accepted and followed, always proved successful.

As intimated above, matrimony might be regarded as the best prescription whenever practicable, under such conditions of mutual attachment, etc., which are necessary to make married life happy. To marry simply for the sake of sexual intercourse is likely to lead to greater unhappiness than can ever be caused by nocturnal emissions.

Illicit sexual intercourse as a substitute for matrimony, is never to be recommended—first, because it is morally wrong, and the physician would take upon himself a fearful responsibility in advising it; and second, because the excesses, which fornication always leads to, have an effect directly opposite to the one desired.

In an admirable lecture—Clinical Lectures and Essays, by Sir James Paget, London, 1875—on sexual hypochondriasis, Sir James Paget says:

"Many of our patients will ask you about sexual intercourse, and some will expect you to prescribe fornication. I would just as soon prescribe theft or lying, or anything else that God has forbidden. If men will practice fornication or uncleanness, it must be of their own choice and on their sole responsibility. We are not to advise that which is morally wrong. even if we have some reason to think a patient's health would be better for the wrong-doing. But in cases before us, and I can imagine none in which I should think differently, there is not ground enough for so much as raising a question about wrong-doing. Chastity does no harm to mind or body, its discipline is excellent; marriage can be safely waited for; and among the many nervous and hypochondriacal patients who have talked to me about fornication, I have never heard one say that he was better or happier for it; several have said that they were worse, and many I know have been made worse."

In all cases of frequent nocturnal emissions, the genital organs should be examined, and whether phimosis exists or not, if the prepuce be long and redundant, circumcision is to be recommended. A very marked varicocele may also render surgical interference desirable. (See last number of Record, p. 339, for hygyenic rules.)

In one severe case of nocturnal emissions, occurring several times every night, Prof. J. H. Pooley made a perineal incision, similar to that for median lithotomy, into the urethra just at the apex of the prostatic gland, and diverted the urine from its natural channel for a time. The result is reported to have been successful, but whether due to the mental or physical effect of the operation may be questioned.

A few words are still necessary with regard to the special complaints made by these patients to their medical adviser, and which have for the most part been enumerated. Trifling as they may appear to him, they should yet be fully explained to them. The lassitude and backache which they experience after an emission, is nothing more than any person of impaired nervous power would feel after a long walk or other exercise. Their loss of memory is purely imaginary. They should be told that the natural condition of the urethra is one of moisture, like the inside of the mouth; that the amount of moisture will vary at different times; that it is especially liable to be increased by the erethism, occurring either with or without the knowledge of the patient during the hours of sleep, and hence is most perceptible in the morning on rising; that it is perfectly natural in men as in the lower animals to have the end of the penis smeared with a clear viscid fluid when under sexual excitement (nature's object probably being to facilitate intromission); that a cloud or sediment will form in the most normal urine when allowed to stand for a few hours, and that no pair of optic ever born, unassisted by the microscope, could discover the presence of semen. But even should it occur, as it sometimes does, that a fluid actually containing spermatozoa, is pressed out from the canal, especially on straining in passing a hard stool, it is nothing more than that overflow from the vesiculæ seminales, to which continent persons in robust health are liable. Spermatozoa left in the canal after such exertion, and particularly after a wet dream during the night, will naturally be washed away by, and be found in the urine the next time it is passed.

The picture that I have given of masturbation and seminal emissions is very different from the one drawn by Lallemand and by the charlatans of the present day, who, in their circulars, represent impotence, diseases of the heart, consumption, paralysis, insanity and idiocy as a few of the consequences of self-abuse, which can only be cured by some nostrum of which they hold the secret. Masturbation is injurious, degrading and beastly enough, not to require to be painted in any colors which are not consistent with truth.

I have taken occasion to make inquiries of some of the most eminent physicians of our insane asylums, as to what extent masturbation should be regarded as a cause of insanity, and they have expressed the decided opinion that it was mental weakness that led to masturbation, and not masturbation that led to mental weakness and insanity. Paget's words on this point are worth quoting :

"You may teach positively that masturbation does neither more nor less harm than sexual intercourse practiced with the same frequency in the same conditions of general health and age and circumstance. Practiced frequently by the very young, that is at any time before or at the beginning of puberty, masturbation is very likely to produce exhaustion, effeminacy, over-sensitiveness and nervousness; just as equally frequent copulation at the same age would probably produce them. Or, practiced every day, or many times in one day, at any age, either masturbation or copulation is likely to produce similar mischiefs or greater. And the mischiefs are especially likely or nearly sure to happen and to be greatest, if the excesses are practiced by those who, by inheritance or circumstances, are liable to any nervous disease—to 'spinal irritation,' epilepsy, insanity or any other. But the mischiefs are due to the quantity, not to the method, of the excesses; and the quantity is to be estimated in relation to age and the power of the nervous system. I have seen as numerous and as great evils consequent on excessive sexual intercourse as on excessive masturbation; but I have not seen or heard anything to make me believe that occasional masturbation has any other effects on one who practices it than has sexual intercourse, nor anything justifying the dread with which sexual hypochondriacs regard the having occasionally practiced it. I wish I could say something worse of so nasty a practice; an uncleanliness, a filthiness forbidden by God, an unmanliness despised by men."

There are other complaints of the sexual hypochondriac, into which we have not the space to fully enter. I refer particularly to those odd caprices which these organs sometimes exhibit under varying mental emotion—even in the most robust and healthy individuals. Perhaps the most frequent complaint is that of too speedy ejaculation in the sexual act, which may take place on attempting intercourse with any woman or with some one woman in particular, especially if the attempt be the first one with her. Here the mind is chiefly at fault; over-anxiety to perform the act well is very likely to lead to its being performed badly. It occurs less frequently in married life than in single, and is a defect which diminishes with age, as old men well know. If art can do anything to hasten its cure, it will be by means already mentioned : circumcision, if the prepuce be long; the cold sound in cases of irritability of the prostate, ergot and the tincture of iron internally: last but not least matrimony, or in lieu of that, some other object in life than sexual gratification.

Cases of absolute impotence in men of good health, and who have not greatly abused their powers, must be rare; I can recall but three or four in many years of practice. These I have treated with almost every remedy which I had ever heard of, but I cannot say that I ever did any good to the patient. Most frequently the impotence is merely an imaginary evil, but, like the boy who did not want to go into the water before he knew how to swim, the patient desires to be satisfied beforehand of his competency. This he cannot do by trial with women of the town; the conditions under which such attempts are made are obviously so different from those of married life as to require no comment.

Finally, the satisfactory accomplishment of the sexual act will be influenced by the merest whim or fancy. One man will be told by a friend some story of his sexual weakness, and, with this in his memory, he too for a time will find himself defective. A coarse word or some personal remark made by a woman may take away all desire for her, while the desire and the power remain the same for others. Excessive desire, especially with gratification long delayed, may also temporarily deprive a man of his power. Ribaud, in Traite de l'Impuissance et de la Sterilite, relates a story of a young Frenchman living in the country, where he was initiated into the pleasures of Venus by a governess, who was a blonde and always wore, when she met him, English boots, corsets and silk dress. When old enough it became desirable for family reasons that he should be married, but he found himself impotent except under the following conditions: the woman must be dressed, must be a blonde, must wear English boots, corsets and a silk dress, in which case his powers were all that could be desired. Under the pretense of giving him a powerful medicine, Ribaud administered a "placebo," which cured him.

This story is here told to show how much a man's powers are influenced by his mental condition, and to enforce the importance of paying attention to the *morale* as well as the *physique* in the treatment of disorders of the genital functions.—*American Practitioner.*

